# Liver-derived Indian hedgehog (Ihh) couples fast-feed transition to thermogenic and metabolic homeostasis

**DOI:** 10.1016/j.molmet.2026.102339

**Published:** 2026-02-26

**Authors:** Raffaele Teperino, Marketa Adamová, Shefa’ Muneer Aljabali, Shruta Pai, Raffaele Gerlini, Irene Paez-Perez, Madlen Matz-Soja, Steffen Heyne, Adelheid Lempradl, Maria Felicia Basilicata, Martin Hrabě de Angelis, Rolf Gebhardt, Erwin Schleicher, Hans-Ulrich Häring, John Andrew Pospisilik

**Affiliations:** 1 Max-Planck Institute of Immunobiology and Epigenetics, Freiburg, Germany; 2 Institute of Experimental Genetics, Helmholtz Zentrum Muenchen, German Research Center for Environmental Health (GmbH), Neuherberg, Germany; 3 German Center for Diabetes Research (DZD) Neuherberg, Germany; 4 Department of Internal Medicine and Department of Clinical Chemistry and Pathobiochemistry, University of Tuebingen, Tuebingen, Germany; 5 Division of Hepatology, Clinic of Oncology, Gastroenterology, Hepatology and Pneumology, University Hospital Leipzig, 04103 Leipzig, Germany; 6 Department of Metabolism and Nutritional Programming, Van Andel Institute, Grand Rapids, MI, USA; 7 Institute of Human Genetics, University Medical Center of the Johannes Gutenberg University Mainz, Mainz, Germany; 8 German Mouse Clinic, Helmholtz Zentrum Muenchen, German Research Center for Environmental Health (GmbH), Neuherberg, Germany; 9 Chair of Experimental Genetics, TUM School of Life Sciences, Technische Universität München, Freising, Germany; 10 Rudolf-Schoenheimer-Institute of Biochemistry, Medical Faculty, University of Leipzig, Leipzig, Germany; 11 Department of Epigenetics, Van Andel Institute, Grand Rapids, MI, USA

**Keywords:** Polycomb Repressive Complex 2, Hedgehog, Hepatokine, Browning, VLDL, Metabolism

## Abstract

**Background & aims:**

Obesity and type 2 diabetes are global health challenges driven by genetic and environmental factors, including diet. While intermittent fasting improves metabolic health, the hepatic mechanisms linking feeding transitions to systemic metabolic regulation remain unclear. We investigated whether Indian Hedgehog (Ihh), a liver-derived hepatokine, coordinates metabolic responses to nutritional transitions.

**Methods:**

We employed genetic and epigenetic tools, including liver-specific deletion of the PRC2 component Eed, to study Ihh regulation. In vivo metabolic phenotyping, thermogenic gene profiling, and Ihh immunoneutralization assessed its function. VLDL-associated Ihh levels were measured and their correlations with metabolic traits were analyzed in humans.

**Results:**

Ihh is induced upon feeding and promotes adipose thermogenesis, enhancing metabolic flexibility. The Ihh locus in hepatocytes resides in a bivalent chromatin state; hepatic Eed deletion derepresses Ihh, conferring resistance to diet-induced obesity and insulin resistance. Immunoneutralization of Ihh reverses this protection, confirming its necessity. Ihh circulates in complex with VLDL. Human Ihh-VLDL levels decline with age and correlate with improved metabolic parameters, including insulin sensitivity, HDL/LDL ratio, and reduced adiposity.

**Conclusions & implications:**

Ihh is a liver-derived, epigenetically regulated hepatokine that links nutrient timing to systemic metabolic control by stimulating thermogenesis and promoting glucose homeostasis. These findings identify Ihh as a key inter-organ signal coupling hepatic chromatin dynamics to energy balance. The age-related decline in circulating Ihh and its strong association with metabolic health suggest that enhancing Ihh signaling may represent a novel therapeutic avenue for obesity and type 2 diabetes.

## Highlights

• Ihh is a novel hepatokine induced during feeding that promotes thermogenesis.
• Liver PRC2 represses Ihh via a poised bivalent chromatin state.
• Hepatic Ihh overexpression protects against obesity and insulin resistance.
• Ihh is secreted in VLDL and correlates with metabolic health in humans.
• Ihh links nutrient timing to inter-organ metabolic coordination.

## 1 Introduction

Glucose homeostasis is a complex and systems-level process aimed at maintaining vital functions and organismal health. Almost every tissue in mammals participates in maintaining glucose homeostasis, either by controlling insulin secretion (as alpha and beta cells in the pancreatic islets), or by responding to circulating insulin with glucose uptake (mostly muscle, adipose tissue and liver). In this context, liver plays a dual role; upon glucose ingestion, it takes up glucose from the circulation and stores it as glycogen; and upon fasting, liver mobilizes these glycogen stores to serve as the primary short-term fuel source to support energy homeostasis via the production of glucose and eventually ketone bodies [1,2].

The liver is also a central regulator of lipid homeostasis. By secreting very low density lipoproteins (VLDL) and internalizing circulating fatty acids and lipoproteins (mostly VLDL remnants), hepatocytes export excess triglycerides - thus preventing liver steatosis - and control fatty acids and cholesterol fluxes between liver, white and brown adipose tissues, muscle and heart [3]. Liver function is therefore tightly bound to organismal metabolism [4,5] and therapeutic concepts already incorporate these mechanisms with the goal of restoring liver function in diabetes and obesity [2].

In addition to transporting lipids themselves, lipoproteins have previously been shown to transport morphogens, a concept conserved from flies to mammals [6,7], and VLDL has been shown to carry biologically active Indian Hedgehog (Ihh) in humans [8]. Bioactive hedgehog ligands are highly lipidated and their post-translational cholesteroylation and acylations are essential for binding and activation of their receptor *Patched* [9]. As a key pathway underpinning development and pattern formation [[10], [11], [12]], activation of canonical and non-canonical hedgehog signaling pathways in adults has been linked to cancerogenesis [13,14] as well as select roles in modifying organismal physiology and metabolism [[15], [16], [17]]. We have for example shown that constitutive activation of canonical hedgehog signaling induces a white adipose selective lipodystrophy in flies and mice [18], and identified a non-canonical signaling cascade that controls muscle and brown fat metabolism in an AMPK-dependent, transcriptional-independent manner [19].

Developmental programs (like Hedgehog signaling mediators) are dynamically regulated throughout development. This developmental control in many contexts is attributed in part to the activity of two related multiprotein chromatin silencing complexes: Polycomb Repressive Complex 2 (PRC2), which catalyzes H3K27 mono-, di-, and tri-methylation [20], and Polycomb Repressive Complex 1 (PRC1), which recognizes H3K27me3 and reinforces silencing via H2A ubiquitination [21]. PRC1 and PRC2 regulate proliferation, differentiation, transcriptional stability, and cancer development [20,[22], [23], [24]]. In terminally differentiated cells, PRC2 prevents inappropriate activation of genomic regions embedded in ‘bivalent’ chromatin—a poised state marked by both active (H3K4me3) and silent (H3K27me3) histone marks [25,26]. While well characterized in development, bivalency in mature cells remains less understood. That said, the relative quiescence of bivalent genes appears crucial for maintaining cell identity [27,28], while the gene content in bivalent regions suggests a role in inducible or pulsatile expression [[29], [30], [31]].

While prior work identified a role for hedgehog signaling in the regulation of brown vs. white fat adipognesis and a novel non-canonical pathway for brown fat and muscle activation, that work left unanswered two key questions constituting a critical knowledge gap towards full recognition and understanding of the Hedgehog pathway in adult metabolic homeostasis. The two key questions – specifically addressed in this study – pertain the endogenous source of adipose-activating hedgehog ligands, and, the physiological contexts underwhich such ligands are working.

Using a combination of human data, mouse functional genetics and molecular analysis, we demonstrate VLDL embedded Ihh (VLDL-Ihh) to be part of a novel liver-to-brown fat hepatokine axis that signals fasted-to-feeding transitions and controls glucose homeostasis. We show that Ihh is embedded in bivalent chromatin and that hyperactivation of the gene (via hepatocyte-specific deletion of PRC2's EED scaffold protein) results in adipose-tissue browning and improved whole-body metabolic homeostasis. We identify a markedly age-associated release of VLDL-Ihh with the fast-feeding transitions, and show through Ihh-specific immunoneutralization experiments that the axis provides an obesity suppressing action under normal conditions. Analysis of small human cohort of healthy volunteers indicate that a fast-feed associated VLDL-Ihh axis is active in humans and that it associates with a young and healthy metabolic state, suggesting the pathway could potentially be leveraged for novel therapeutic strategies.

## 2 Material and methods

### 2.1 Reagents

Unless otherwise stated, all chemicals and reagents were obtained from SIGMA.

### 2.2 Animal husbandry

All mice were maintained under controlled temperature (22 °C) and on a 12hr light, 12hr dark schedule (light on 6:00–18:00). Food and water were available ad libitum unless otherwise stated. All mice were weaned at 3 weeks of age onto a standard chow (SDS RM3, Essex, UK or V1185-300 MZ-Ereich, ssniff, Germany).

For High-Fat-Diet studies, mice were fed ad libitum on a standard, irradiated High-Fat-Diet (D12492 - 60% Kcal from fat – Research Diets Inc.) for 5 months starting at 6 weeks of age.

For thermoneutrality and cold exposure experiments, 12 wk-old animals were fed ad libitum and transferred to a temperature controlled chamber (TSE System) with 12hr light, 12hr dark schedule. For thermoneutrality, mice were exposed to 28**°**C for 4 weeks; body weight measured before and after the exposure; and one week of indirect calorimetry measurement at the end of the experiment. For cold exposure, mice were kept at thermoneutrality for one week before being exposed to 8**°**C for 6hr. Core body temperature was measured using a rectal probe longitudinally every 2 h interval during the experiment. At the end of the experiment, mice were sacrificed by cervical dislocation and tissues collected for further downstream analyses.

LEedKO mice were generated as previously described [29] and genotyped using established primers and PCR amplification protocols [29]. Skeletal Muscle, white adipose and brown adipose tissue-specific Smoothened (Smo) knockouts were generated by crossing Smo-floxed (Smo^fl/fl^ - *Smo*^tm2Amc^/J mice obtained from Jackson Laboratories) with Cre-transgenic mice expressing the enzyme under the Mck (Muscle Creatin Kinase), aP2 (Fatty Acid Binding Protein 4) or Ucp1 (Uncoupling Protein 1) promoter, respectively.

All animal studies were performed with the approval of the local authority (Regierungspräsidium Freiburg, Germany) under license number 35.9185.81/G-10/94. Due to ethical constraints, the sexual dimorphism of murine metabolic homeostasis and the need to reduce the total number of mice, only male mice were used for the described phenotypic analyses.

### 2.3 Glucose and insulin tolerance test

Wild-type and LEedKO mice (16 week old) were over-night fasted and an oral glucose tolerance test (OGTT) was performed by administering 2 g/kg glucose by oral gavage. Tail-vein blood was collected to measure glucose and insulin levels. Insulin was measured using an ultrasensitive insulin ELISA kit (Mercodia), according to the manufacturer's recommendations. For insulin tolerance tests mice were fasted for 6 h (from 9am to 2pm) and i.p. injected with insulin (Humulin® - Eli Lilly 0.75U/Kg). Tail-vein blood was collected to measure glucose levels.

### 2.4 Indirect calorimetry

To measure basal metabolic rate, 10- to 14-week-old animals were singly housed in a home-cage indirect calorimetry system (TSE Systems). Animals were monitored over a 6 day period and fed an ad libitum chow diet or 60% HFD according to experimental designs. Data from the first day were discarded to reduce variation introduced by acclimatization. Data from consecutive days were binned in 3hr intervals and treated as technical replicates.

Oxygen consumption (VO2) and CO2 production (VCO2) data have been normalized to lean mass, measured before the indirect calorimetry experiment by non-invasive nuclear magnetic resonance spectroscopy with a Minispec NMR analyser (Brucker Optics), according to the manufacturer's instructions.

### 2.5 Euglycaemic hyperinsulinemic clamp

Euglycaemic hyperinsulinemic clamp experiments were performed on 12 wk old, WT and LEedKO mice as previously described [18,19]. Between 30 and 60 min, four blood samples were collected for calculation of insulin-mediated suppression of endogenous glucose appearance rate, a marker of hepatic glucose production (HGP). HGP during insulin-stimulated condition was calculated by subtracting the glucose infusion rate (GIR) from the rate of disappearance. To determine glucose utilization of individual tissues, mice were injected with ^3^H-2-deoxyglucose (Perkin Elmer) through the intrafemoral catheter 1 h before completion of the infusion procedure. Tail blood was sampled at 5, 10, 15, 20, 30, 45, and 60 min after the injection to determine the time course of ^3^H-2 deoxyglucose disappearance. The ^3^H-2 deoxyglucose-6-phosphate content was determined from NaOH hydrolysed tissues by the Somogyi procedure.

### 2.6 Hedgehog immunoneutralisation

For immunoneutralisation of circulating Hedgehog peptides, HFD-fed LEedKO mice were treated for 16weeks with 1 mg/kg (weekly ip. injections) of the Hh neutralizing antibody 5E1 (DSHB – University of Iowa) [32] or its isotype IgG control. Food intake, blood glucose and core body temperature were monitored during the experiment without any significant difference (data not shown).

### 2.7 Clinical chemistry

Blood samples for clinical chemistry analyses were collected in Li-heparin coated tubes after overnight food withdrawal and analyzed using an AU480 autoanalyzer (Olympus, Germany).

### 2.8 Histology (H&E and IHC)

For tissue sections, hematoxylin and eosin (H&E) staining and Immunohistochemistry (IHC) were performed on 1.5 μm paraffin sections of tissues fixed in 4% phosphate-buffered formalin. For H&E, the slides were deparaffinized in xylene and rehydrated in alcohol (from 100 to 50% EtOH solutions). Hematoxylin was applied for 2 min and then slides were washed with warm tap water until tissue staining turned blue. Then slides were placed in Eosin for 1 min and washing with tap water was performed for 5 min. Alcohol dehydration was carried out for 20 s in each solution starting with 30 % à 70% à 80 % à 96 % x 2 à 100% x 2. Then the slides were transferred into xylene for 5 min, mounted with Pertex. Then they were incubated at 60**°**C. Images were acquired at an Olympus BX43 Microscope.

IHC was performed on deparaffinized and rehydrated slides. Antigen-retrieval treatment (Citrate, EDTA or Proteinase K) was performed according antibody specifications. Hydrogen peroxide was used for 10 min for the inhibition of endogenous peroxidase. Washing was performed with TBS-T buffer (10x TBS Buffer diluted in dH_2_0 + 0.1 % Tween 20) and then goat or rabbit serum, diluted in TBS-T, was used as a blocking solution for 30 min. Primary antibody incubation was performed overnight at 4**°**C in a humidified chamber according to antibody specifications. Controls were performed without the application of primary antibodies. On the next day, the slides were washed with TBS-T and incubated with the appropriate biotinylated secondary antibody for 30 min at room temperature. After washing, Streptavidin Peroxidase was used for 15 min to aid detection by increasing the binding sites on the secondary antibody. Next, the slides were washed with TBS-T and 200 μL of the Signal Stain DAB Substrate Kit [15:1000] were used for 6 min for substrate precipitation. Hematoxylin was used for counterstaining of the nuclei for 10 s before alcohol dehydration. Then the slides were transferred into xylene for 5 min and mounted wit Pertex mounting medium and a coverslip. Then they were incubated at 60**°**C. Images were acquired at an Olympus BX43 Microscope.

### 2.9 Antibody specifications for IHC

Rabbit Polyclonal **anti-IHH** - abcam Cat #ab39634 (1:1000).

Mouse monoclonal **anti-PanCK [clone AE1/AE3]** - Invitrogen Cat #18–0123 (1:100).

Rabbit Polyclonal **anti-Cytokeratin WSS** - Dako Cat #Z0622 (1:700).

**Anti-H3K27me3** - Gift from Thomas Jenuwein already used in [29].

Rabbit Polyclonal **anti-Ucp1** - Invitrogen Cat #PA1-24894 (1:500).

### 2.10 Western Blot

Tissues were homogenized and lysed in RIPA buffer (50 mM Tris pH 7.6, 150 mM NaCl, 1 mM EDTA, 1% Triton X-100, 1% sodium deoxycholate, 0.1% SDS) containing protease and phosphatase inhibitors (Roche). Lysates were cleared by centrifugation at 4 °C at 16,000 g for 30 min. Protein concentration in the supernatant was determined using the BCA Protein Assay Kit (Pierce). 20–30 μg of proteins were resolved by SDS-PAGE and transferred to PVDF membranes (GE Healthcare). Membranes were blocked with 5% BSA in Tris-buffered saline containing 0.2% Tween-20 (TBS-T), and incubated with primary antibodies at 4 °C over night. Antigen-specific binding of antibodies was detected with SuperSignal West Femto and Pico Kits (Pierce) using a ChemiDoc XRS Imager (Bio-Rad). Image analysis was performed using Image Lab Software Version 3.0.1 (Bio-Rad).

### 2.11 Antibody specifications for Western Blot

Rabbit Polyclonal **anti-IHH** - abcam Cat #ab39634 (1:1000).

Rabbit monoclonal **anti-Gli1** - Cell Signalling Technology Cat. #3538 (1:1000).

**Anti-H3K27me3** - Gift from Thomas Jenuwein already used in [29].

Rabbit Polyclonal **anti-Ucp1** - Invitrogen Cat #PA1-24894 (1:1000).

Mouse monoclonal **anti-Hsp90** [clone D7a] - Sigma/Merck Cat #05–594 (1:1000).

Rabbit monoclonal anti-H3 [clone D1H2] - Cell Signalling Technology Cat. #4499 (1:1000).

### 2.12 RNA isolation and real-time PCR

Total RNA was extracted using TRI Reagent (SIGMA) and was reverse transcribed into cDNA using commercially available kits (Applied Biosystems). qPCR reactions were performed a 7500HT Fast Real-Time PCR System (Applied Biosystems). Post-amplification melting curve analysis was performed to check for unspecific products and primer-only controls were included to ensure the absence of primer dimers. For normalization threshold cycles (Ct-values) were normalized to within each sample to obtain sample-specific ΔCt values (= Ct gene of interest - Ct housekeeping gene Rplp0). 2-ΔΔCt values were calculated to obtain fold expression levels, where ΔΔCt = (ΔCt treatment - ΔCt control).

Primers sequences.

**Shh**.

For. CCAAAAAGCTGACCCCTTTAG.

Rev. ATCCTTAAATATGATGTCGGGGT.

**Dhh**.

For. GGAGAGGGAGGGGGAGGGAGAAAAT.

Rev. TTAGCCTCTCCCCCAGTGCTTCAGC.

**Ihh**.

For. GCGCCGACCGCCTCATGACC.

Rev. TCTGATGTGGTGATGTCCACCG.

**Rplp0**.

For. TGCACTCTCGCTTTCTGGAGGGTG.

Rev. AATGCAGATGGATCAGCCAGGAAGG.

### 2.13 RNA-seq and data analysis

Trizol-purified RNA was poly(A)-enriched, and libraries were prepared with a TruSeq Sample Prep v2 kit (Illumina) and sequenced on a HiSeq 2500 (Illumina). Read mapping and differential expression analysis was performed using the A.I.R (Artificial Intelligence RNA-Seq) software from Sequentia Biotech with the following pipeline: *BBDuk* (reads trimming - http://jgi.doe.gov/data-and-tools/bbtools/bb-tools-user-guide/bbduk-guide/), *STAR* (reads mapping to the mouse genome GRCm38 [ENSEMBL] - https://github.com/alexdobin/STAR), *featureCounts* (gene expression quantification - http://bioinf.wehi.edu.au/featureCounts/), and *NOISeq* (statistical analysis of differentially expressed genes - http://bioinfo.cipf.es/noiseq/doku.php). Compared to other methods to calculate differential expression, *NOISeq* is a data adaptive non-parametric method specifically designed to account for high variability across replicates and genes with low expression level [33]. Heatmap and PCA analyses were performed with the web-application ClustVis using default parameters [34]. KEGG analysis was performed with DAVID using default parameters [35].

### 2.14 Chromatin segmentation analysis

Publicly available wild-type mouse liver ENCODE and wild-type mouse hepatocytes DEEP (German Epigenome Project) ChIP-Seq datasets (H3K4me1, H3K4me3, H3K9me3, H3K27me3, H3K27Ac, H3K36me3) were downloaded from the respective portals and re-analyzed according to ENCODE guidelines [36]. BigWig files have been visualized using the Integrated Genome Viewer software (version 2.3.92). We used EpicSeg for chromatin segmentation [37]. Chromatin states were assigned to genes according to (1) the maximum single state coverage over the genebody and (2) and the chromatin state at the TSS (fold enrichment >2.0 and *p-value* < 10^−4^). Assignments were only done to the ‘‘basic’’ subset from Gencode M9 and to genes with biotype ‘‘protein_coding’‘,’‘lincRNA’’ or ‘‘antisense’‘.

### 2.15 Ex-vivo seahorse assay

Respiratory activity in WT and LEedKO scWAT was measured using an XF24e instrument (Seahorse Bioscience – Agilent) as previously described [38] with the following modifications. Briefly, freshly excised scWAT was cleaned of non-adipose material, cut into small pieces (∼10 mg) and placed in individual wells of a 24-well islet capture microplate covered by one islet capture screen. Fatty acid oxidation was stimulated by Isoproterenol treatment (1 mM – SIGMA) and OCR data normalized to tissue genomic DNA content.

### 2.16 Indian hedgehog ELISA

Serum was obtained from young (12wk-old), old (48wk-old) and HFD-fed (22wk-old) WT and KO animals after 24hr fasting or 24hr fasting followed by 1hr refeeding. Circulating Ihh levels were measured by ELISA (SED116Mu – Cloud-clone corporation, Houston Texas USA) according to manufacturer's instructions.

### 2.17 Human studies

We included 37 healthy male volunteers in the study with an age ranging from 25 to 72 years and a BMI ranging from 28 to 48 kg/m2. Participants were included when they fulfilled at least one of the following criteria: a family history of type 2 diabetes, as having at least one first degree relative with type 2 diabetes or a BMI >28 kg/m^2^. Exclusion criteria were impaired renal function and previous diagnosis of diabetes or the presence of a severe critical mental or physical illness. Participants previously receiving lipid lowering drugs were also excluded. None of the participants was on any glucose lowering medication. Clinical characteristics are presented in Table 1. All participants underwent a screening visit with medical history and gave clinical consent. Blood samples were withdrawn after overnight fasting. The study was approved by the Institutional Ethics Committee of the Medical Faculty of Tübingen (422/2002) and conducted in accordance with the declaration of Helsinki.

**Table 1:** Anthropometric and clinical data of the studied cohort interquartile range.

Trait (N = 37)	Median	Interquartile range (25–75%)
**Age (years)**	49	33–63.25
**Sex (f/m)**	0/37	
**BMI (kg/m^2^)**	29.6	27.6–32
**Total cholesterol (mg/mL)**	185	174–210
**HDL cholesterol (mg/mL)**	44	30–54.5
**Triglyceride (mg/mL)**	108	75–157
**Fasting glucose (mM)**	5.5	5.17–5.83
**Insulin sensitivity (ISIMATS)**	11.4	6.47–17.47


	Age ≤40 (N = 14)		Age >40 (N = 23)	
	Median	Interquartile range	Median	Interquartile range
**Age (years)**	31	27–35	59	47–65
**BMI (kg/m^2^)**	39.9	24.5–33.4	31.4	28.2–36.3

### 2.18 Determination of VLDL-bound ihh

VLDL was isolated from EDTA plasma obtained by venipuncture of the study participants. VLDL fraction was separated from the HDL and LDL fractions by ultracentrifugation. For this separation, 1 ml sodium chloride solution (1.006 g/ml) to 1 ml human plasma was added. Subsequently, ultracentrifugation at 40,000 rpm (817,480 g) and 10 °C was performed for 18 h using a preparative ultracentrifuge (Optima; Beckman Coulter, Palo Alto, CA, USA). Afterwards, the top layer (VLDL fraction) was removed for further analyses. Triacylglyceride concentrations were determined by the clinical chemical analyzer Advia Chemistry XPT (Siemens, Eschborn, Germany) and ihh was measured with an immunoassay obtained from Cloud-Clone Corporation (Houston, Texas, USA). The coefficient of variation was 2.4% within run and 4.6% from day to day indicating very good precision of the assay.

### 2.19 Western Blot

Pure lipoprotein fractions (VLDL, LDL and HDL) were obtained from pooled human plasma by high speed ultracentrifugation and subsequent washing with saline and concomitant concentration by further ultrcentrifugation [39]. Deglycosylation was performed with a 10 μl deglycosylation mix containing both O- and N-Glycanase as described by the manufacturer's instructions (#P6044S New England Biolabs - NEB). Deglycosylated or untreated lipoproteins (50 μl) together with 10 μl Lämmli buffer were applied to 7.5–19% sodium dodecyl sulfate polyacryl-amide gradient gel electrophoresis. Thereafter proteins were transferred to a nitrocellulose membrane by semidry electroblotting. Immunodetection was performed using a mouse monoclonal antibody the full-length recombinant human Ihh (ab39634) (Abcam, Cambridge, U.K.). Separated protein were visualized by the Odysee method. Human liver extracts were used as positive control alone or as 1:50 spike-in.

### 2.20 Cell culture and transfection

Shh-LIGHT2 experiments were performed as previously described [40]. Briefly, Shh-LIGHT2 cells were starved overnight in 0.5% serum containing medium and stimulated for 48 h with the recombinant sonic Hh (Shh) protein (200 ng/ml – R&D Systems, Minneapolis, MN) or WT and KO mouse serum (10% in growing medium), obtained by terminal mouse bleeding after 24hr fasting (Fast) or 24hr fasting followed by 1hr chow-diet refeeding. Serum delipidation was performed as previously described [41]. Hh immunoneutralization was obtained by one week treatment of mice with 1 mg/kg (daily ip. injections) of the Hh neutralizing antibody 5E1 (DSHB – University of Iowa) [32] or isotype IgG controls. Hh signaling activities in Shh-LIGHT2 cells were measured using the Dual-Luciferase Reporter Assay System (Promega, Madison, WI). Signal intensities were normalized to Renilla luciferase (Shh-LIGHT2 cells) as described [42].

### 2.21 Statistical analysis

Data are expressed as mean ± standard error of the mean (SEM) unless otherwise specified. Statistical significance was tested by Student's t-test or ANOVA where appropriate. Correlations analyses were done using the Correlation Matrix analysis tool of GraphPad Prism 10 (version 10.4.2). The correlation matrix (and the single correlations) results report the *Pearson r* correlation coefficient and the single nominal *p*-values, based on the null hypothesis that the true population correlation coefficient is zero. To account for multiple correction, FDRs have been further calculated from the stack of *p*-values using the Benjamini-Krieger-Yekutieli method with a 10% Q threshold. All figures and mouse statistical analyses were generated using Prism 10 (GraphPad). All reported p-values are two-tailed unless stated otherwise. p < 0.05 was considered to indicate statistical significance.

### 2.22 Use of large language models (LLMs)

LLMs have neither been used for writing not editing this manuscript.

## 3 Results

### 3.1 Circulating Ihh is a VLDL-embedded hormone that tracks fast-feeding cycle, age and metabolic health

We previously showed intracellular Hh-signaling to be a potent regulator of thermogenic adipose tissue differentiation and function [18,19]. Those studies left it unclear, however, which endogenous ligand might mediate pathway activation in vivo. Under the hypothesis that the relevant ligand might be circulating we searched for evidence of canonical hedgehog ligands in serum. Whereas Hh-signaling is classically known as a paracrine/morphogen system, cell culture and *Drosophila* studies have indicated that canonical Hh ligands can be secreted in alternate forms [43,44], and proteomics specifically demonstrated that circulating lipoprotein-bound Ihh can be found in human plasma [8].

**Figure 1:**
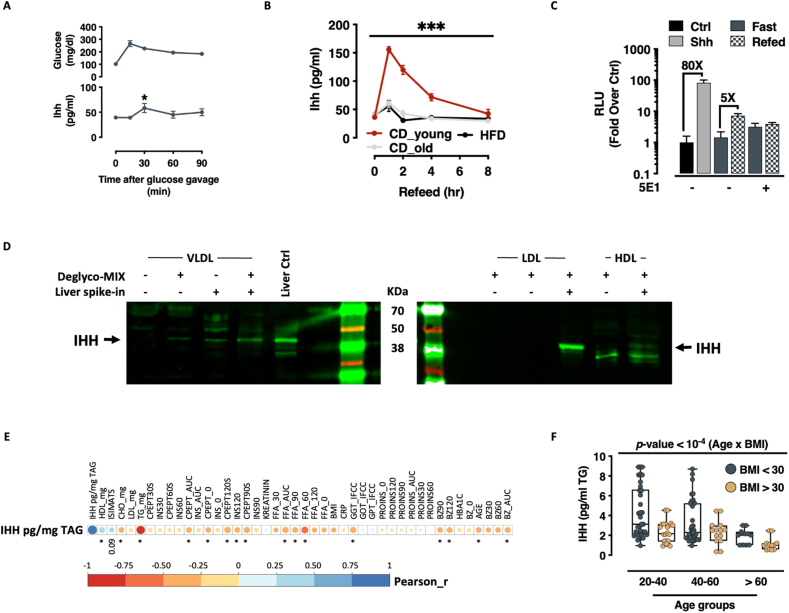
**- Circulating Ihh is a VLDL-embedded hormone that tracks fast-feeding cycle, age and metabolic health. A.** Plasma glucose (top) and Ihh (bottom) excursions during an oral glucose tolerance test (data expressed as mean ± SEM; n = 6 ∗ = *p-value* < 0.05, 2-tailed T-Test) **B.** Plasma Ihh excursions upon fast-refeeding transitions in chow- or HFD-fed young mice or moderately aged mice (data expressed as mean ± SEM; n = 6 *adj.p-value* < 10^−4^, 2-way ANOVA with Geisser-Greenhouse correction and Tukey's multiple comparisons test). **C.** Gli-luciferase reporter activity in Shh-LIGHT2 cells treated with Shh (positive control), mouse plasma upon fast/re-feeding experiment and fast/re-feeding mouse plasma immunoneutralized with the α-Hh antibody 5E1 (data expressed as mean ± SEM; n = 6). **D.** Western blot detection of Ihh from human purified lipoproteins. A deglycosylation treatment (see methods for details) has been performed to remove lipoproteins-bound glycan structures and enhance signal detection. Human liver extracts have been used undiluted as positive control (Liver Ctrl) or diluted 1:50 as internal spike-in control (Liver spike-in) **E.** Pearson-based correlation matrix between VLDL-Ihh and parameters of human glucose and lipid homeostasis. (Pearson correlation analysis - ∗ = significant correlation, *p*-value <0.05) **F.** Circulating VLDL-associated Ihh levels in humans stratified by BMI and age-groups. Significance calculated by 1-way ANOVA with Brown-Forsythe and Welch corrections.

We screened metabolically relevant plasma samples for Ihh dynamics in wild-type C57Bl6/J mice. Plasma Ihh showed evidence of potential inducibility upon low-dose oral glucose tolerance testing (Figure 1A). Interestingly, we found that fast-refeed protocols, in particular, were able to generate significant responses, increasing plasma Ihh levels approximately 4-fold (Fig. 1B). Equally notable, we observed a highly blunted (less than 1-fold compared to fasting state) Ihh secretory response in animals exposed to chronic high fat diet (Fig.1B), and also in chow fed animals that had reached middle age (>24 weeks) (Fig.1B). This novel fast-refeed Ihh response was transient, lasting approximately 4–8 h. We next tested whether the Ihh detectable in fasted and fed plasma samples was biologically active. We applied the samples to a cellular reporter assay for Hh signaling that measures induction of *Gli2* transcription, a hedgehog-reponsive gene. Indeed, fast-refeed plasma samples triggered a robust (5-fold) *Gli2* transcriptional response (Fig.1C). Importantly, this response was absent in fast-refeed samples obtained from animals injected with the hedgehog-immunoneutralizing antibody 5E1 (Fig.1C). These data suggest plasma Ihh levels as a new dynamic readout of the fast-feeding transition.

### 3.2 Circulating Ihh in humans is associated with metabolic health

**Supplementary Figure 1:**
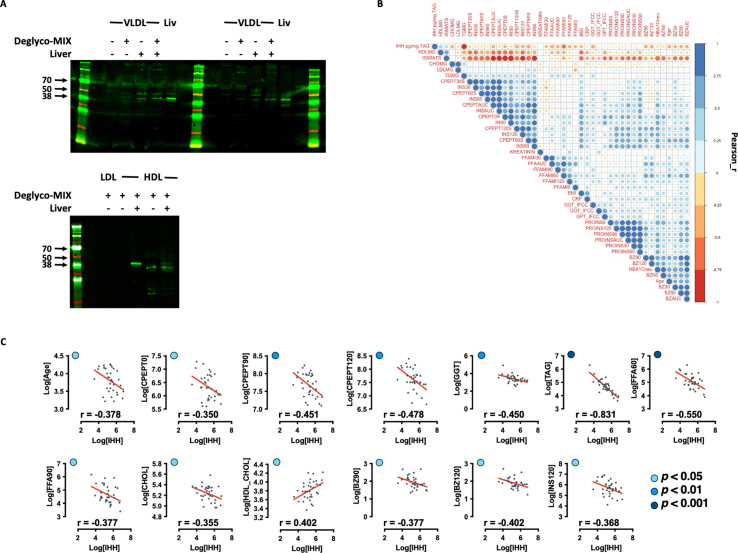
Related to figure 1. **Circulating Ihh is a VLDL-embedded hormone that tracks fast-feeding cycle, age and metabolic health. A.** Original blot figure related to figure 1D. **B.** Full representation of the correlation matrix extracted in figure 1E **C.** Individual plots of the significant correlations highlighted in figure 1E.

In humans, lipoproteins control nutritional homeostasis by compartmentalizing triglyceride- and cholesterol-stored energy for coordinated utilization by the periphery [3]. In order to be active, hedgehog ligands have also been previously shown to be both cholestrolated, and palmitoylated, a double modification that makes active hedgehog ligands highly lipophilic. Consistent with these ideas [8], we found Ihh immunoreactivity was highly and specifically enriched in the VLDL lipoprotein fraction of human plasma (Figure 1D and Suppl. Fig. S1A) after enzymatic digestion of the lipoprotein-bound glycan structures [45] (see methods and figure legend for details). Since VLDL particles are responsible for transferring energy to the periphery (eg. muscle/adipose) these findings (and the fast-refeed dynamics above) suggested that Ihh might be involved in signaling the influx of external nutrient availability from the liver to the periphery.

To test this idea, we leveraged a cohort of 37 healthy male volunteers ranging in age from 25 to 72 years, and BMI from 24 to 48 kg/m^2^ (Table 1), and measured VLDL-associated Ihh after overnight fasting as well as during oral glucose tolerance tests (OGTT). Importantly, and similar to the findings in mouse above, we found that fasting VLDL-Ihh correlated strongly and inversely with human age (Figure 1E–FandS1**B**). Ihh levels were increased relative to total VLDL triglycerides suggesting that concentration increases within any given VLDL particle. Inverse associations were also found with blood sugar (BZ); Insulin (INS); C-Peptide (CPEPT) and other relevant measures of glucose homeostasis, as well as multiple parameters related to lipid metabolism, including Free Fatty Acid levels (FFA) and total Cholesterol (CHOL) (Fig. 1E and S1B–C). VLDL-Ihh was directly correlated with ‘healthy’ HDL-Cholesterol (r^2^ = 0.16), and, a non-significant association was observed with Matsuda Whole Body Insulin Sensitivity Index (ISIMATS – r^2^ = 0.08; p-value = 0.09) (Fig. 1E and S1B–C). Collectively, these signals all pointed towards an association between plasma VLDL-Ihh levels and metabolic health in humans. Thus, VLDL-Ihh is a novel endocrine signal, associated with fast-refeed transitions, age, and metabolic state.

### 3.3 Ihh is a transcribed, epigenetically sensitive bivalent gene

**Figure 2:**
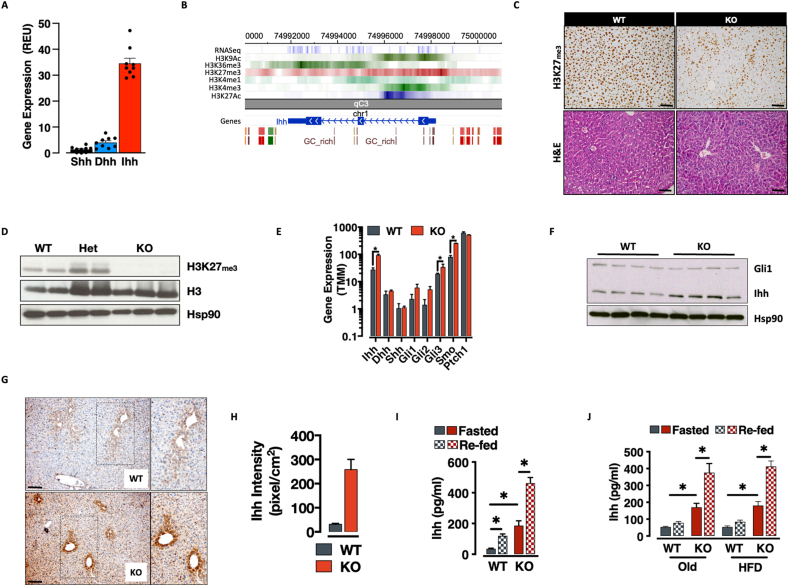
**Ihh is a transcribed, epigenetically-sensitive bivalent gene. A.** qRT-PCR-based relative gene expression of the three Hedgehog ligands in mouse liver (Data is expressed as mean ± SEM of Log10 Relative Expression Unit over an internal standard. n = 8). **B.** WashU Genome Browser snapshot of the Ihh locus in murine adult liver. **C.** Immunohistochemistry (IHC) representation of PRC2 loss of function in the vast majority of the hepatocytes compartment (top) and H&E-based representation of liver morphology (bottom) in WT and Eed-PRC2 knockout mice. **D.** Western-blot representation of PRC2 loss-of-function in LEedKO hepatocytes. **E-H.** RNA (RNA-seq based data. ∗ = *adj.p-value* < 0.05 - **E**), protein (**F**) and IHC-based (**G-H**) representation of the Ihh de-repression in Eed-PRC2 knockout hepatocytes. **I-J.** Plasma Ihh concentration in WT and LEedKO mice upon fast-refeeding in young (**I**), old and HFD-fed mice (**J**) (data expressed as mean ± SEM; n = 6 ∗ = *p-value* < 0.05, 2-tailed T-Test).

**Supplementary figure 2:**
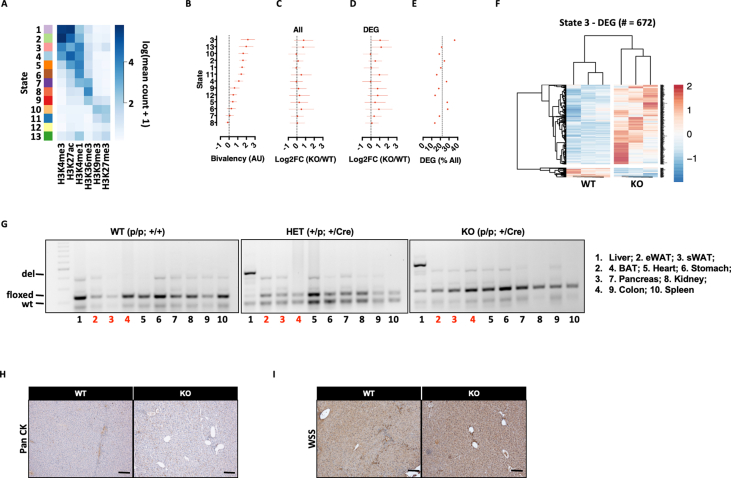
related to figure 2. **Validation of the Eed-PRC2 knockout model. A.** EpiCSeg chromatin-state segmentation of mouse hepatocytes epigenome. **B.** Quantified bivalency (as combined H3K4me3 and H3K27me3) across the identified 13 chromatin states. **C-D.** Quantification of the effect of Eed-PRC2 knockout on “All” expressed genes in hepatocytes (**C**), and on genes differentially expressed between KO and WT animals (**D**). **E-F.** Quantification of the specific effect of Eed-PRC2 in de-repressing bivalent promoters (>40% of derepressed genes in Eed-PRC2 knockout are bivalent (State 3 - **E**), and representative Heatmap of the derepression of state 3 genes upon Eed-PRC2 knockout (**F**). **G.** Representative agarose gel result of a LEedKO genotyping PCR. **H–I.** Immunohistochemistry (IHC)-based analysis of ∗liver morphology and epithelial organization in WT and LEedKO mice. PanCK = Pan Cytokeratin (Mouse mAb AE1/AE3), WSS = Cytokeratin Wide Spectrum Screening.

VLDL particles are produced in the liver by hepatocytes. Interestingly, among the endogenous ligands of the Hedgehog signaling pathway, Ihh is the only one specifically transcribed by the gastro-intestinal tract (not shown) and the most abundantly transcribed in the liver (Figure 2A). In order to better understand Ihh expression control we explored liver and hepatocyte epigenome data mapped by the ENCODE consortium (Fig. 2B) and the German Epigenome Project (Supp Fig. S2A–B - DEEP) [37] respectively. Using the *EpicSeg* algorhithm, we mapped promoter-associated chromatin domains genome-wide and clustered genes based on promoter-associated chromatin modules (from the most bivalent to the least – Supp. Fig. S2A–B and Supp. Table S1). Interestingly, given its high detectable transcript and protein expression, we found the Ihh locus (and promoter in particular) embedded in a bivalent chromatin (H3K4me3^+^, H3K27me3^+^) associated with additional active (H3K9Ac^+^ and H3K27Ac^+^) and elongation marks (H3K36me3^+^) (Fig. 2B). These data suggested that Ihh exhibits inducible gene expression dynamics.

### 3.4 Eed/PRC2 deletion triggers ihh overexpression and secretion

To genetically amplify VLDL-Ihh in vivo, we turned to mature hepatocyte-specific Eed/PRC2 knockout mice (LEedKO) that we had previously generated in the lab. Based on literature and our own previous work with Ezh2 and Eed conditional alleles [29,46], we knew that Eed/PRC2 knockout should specifically derepress bivalent loci and produce modest overexpression of Ihh, while leaving all critical enhancer and promoter mechanisms and secretory control intact.

LEedKO mice were generated by crossing hepatocyte-specific Albumin-*Cre* transgenic mice to a line bearing a conditional *Eed* allele, both in congenic C57Bl6/J contexts (Supp Fig. S2G) [47]. LEedKO mice were born at expected Mendelian ratios. LEedKO animals showed a near complete lack of hepatocyte H3K27me3 immunoreactivity, confirming the Eed deletion and loss of PRC2 function (>90%) (Figure 2C–D). Interesting given the robust deletion, liver morphology was normal in LEedKO mice with no evidence of altered structure, fibrosis, or immune cell infiltration (Fig.2C and S2H-I). Purified hepatocyte transcriptome analysis validated the model and the chromatin segmentation analysis, revealing de-repression specifically of bivalent genes (Supp. Fig. S2C–F and Supp. Table S1), and importantly, Ihh (Figure 2E, S3A). Thus, we generated hepatocyte-specific Eed/PRC2 loss-of-function mutants, with associated Ihh over-expression.

**Supplementary figure 3:**
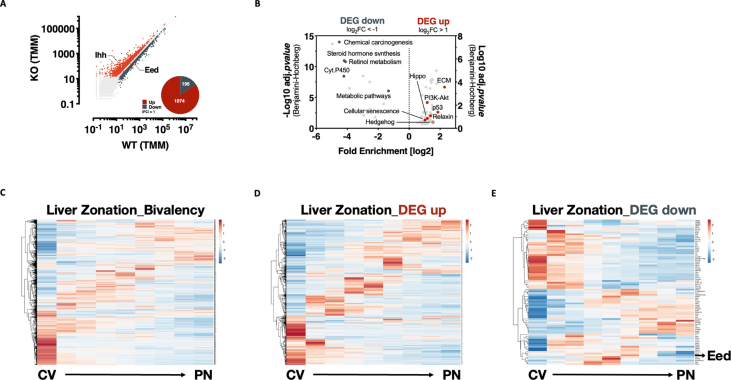
related to figure 2. **LEedKO hepatocytes transcriptomic analysis. A.** Scatter plot representation of the RNA-seq data in WT and LEedKO hepatocytes. Upregulated genes in red, downregulated genes in grey. The cake plot represents the global transcriptional de-repression upon hepatocytes Eed-PRC2 knockout. **B.** KEGG-based pathway analysis of Eed-PRC2 controlled genes in hepatocytes. **C-E.** Representative heatmap indicating the zonated expression of bivalent genes (**C**), and genes controlled by Eed-PRC2 (**D-E**). CV = Central Vein; PN = Portal Node.

Pathway enrichment analysis of differentially expressed genes revealed dampened xenobiotic metabolism and enriched metabolic, oncogenic and developmental pathways in LEedKO hepatocytes (Supp. Fig. S3B and Table S2). As indicated, RNA and protein analyses validated the intervention as a model of Ihh overexpression. Ihh was upregulated on the transcript level in LEedKO hepatocytes (Figure 2E, S3A). Western blotting and Immunohistochemistry (IHC) validated Ihh overexpression (Figure 2F–H) and showed that increased Ihh protein was found across the hepatocyte compartment but mainly in hepatocytes surrounding the portal node (Figure 2F–G). This zonated protein expression pattern was normal, simply enhanced, with Ihh also biased towards the portal node in wild-type animals (Fig. 2F). Notably, this appeared to be a characteristic of most bivalent genes (Supp. Fig. S3C) and derepressed Eed-repressed genes (Supp. Fig. S3D–E) suggesting that relative differences in H3K27me3-dependent silencing potentially underpin the transcriptional patterns that define zonation. Importantly, plasma analysis not only show circulating Ihh levels ∼4-fold increase in LEedKO animals (Fig.2I), the genetic intervention eliminated the previously observed age- and HFD-associated blunting of the fast-refeed Ihh secretory dynamics, with older and HFD-treated LEedKO animals consistently exhibiting a protected or “youthful” Ihh responses (Fig.2J). These data identify LEedKO animals as a model of exagerated hepatic Ihh secretory response and demonstrate that Eed/PRC2 limits Ihh expression and release from hepatocytes. The data therefore indicate hepatic PRC2 function contributes to age- and HFD-associated decline in the normal Ihh secretory response.

### 3.5 Hepatic Ihh improves energy metabolism and confers resistance to diet-induced obesity

**Figure 3:**
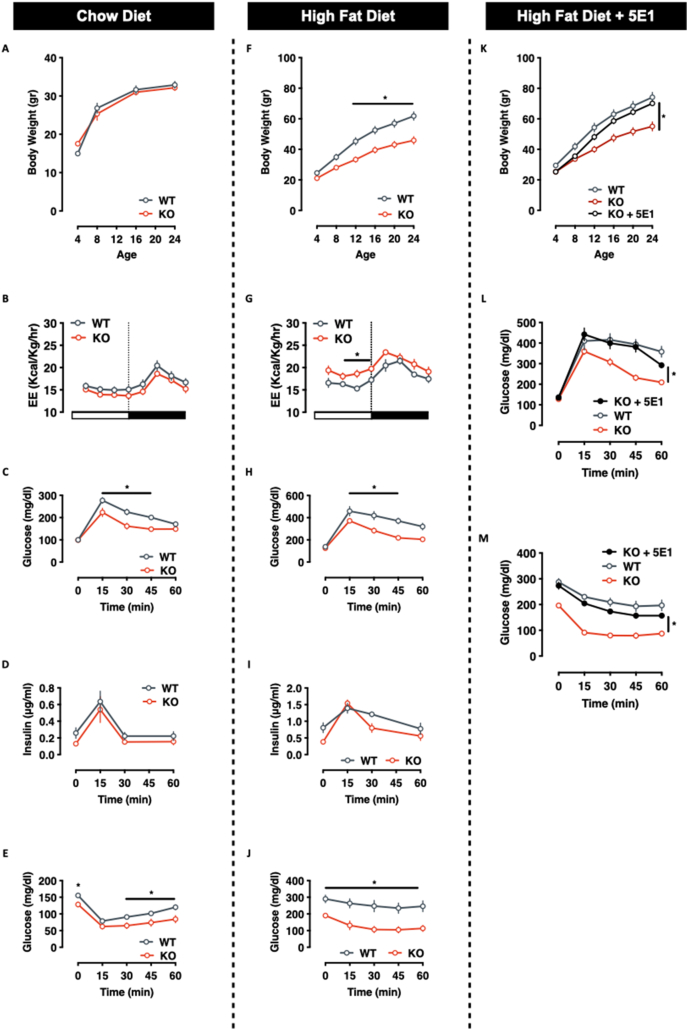
**Eed/PRC2 ablation improves energy metabolism via Ihh. A-E.** Metabolic homeostasis of WT and LeedKO mice on chow diet. (**A**) Body weight, (**B**) Energy expenditure, (**C**) Glucose tolerance (ipGTT), (**D**) in vivo glucose-induced insulin secretion, (**E**) Insulin sensitivity (ipITT). **F-J.** Metabolic homeostasis of WT and LeedKO mice on High-Fat Diet. (**F**) Body weight, (**G**) Energy expenditure, (**H**) Glucose tolerance (ipGTT), (**I**) in vivo glucose-induced insulin secretion, (**J**) Insulin sensitivity (ipITT). **K-M.** Body weight curve (**K**), glucose tolerance (**L**) and insulin sensitivity (**M**) in HFD-fed WT, LEedKO and LEedKO mice pre-treated with the soluble α-Hh antibody 5E1. Data expressed as mean ± SEM; n = 8–12 male mice/genotype; ∗ = *p-value* < 0.05).

LEedKO mice developed and grew normally. Under standard conditions, LEedKO mice show no or mild differences in body weight and energy expenditure (Figure 3A–B), with improved glucose tolerance and insulin sensitivity (Figure 3C–E). Interestingly however, Eed/PRC2 ablation markedly improved the metabolic response of LEedKO animals to a chronic HFD challenge. LEedKO mice were resistant to diet-induced weight gain (Figure 3F) showing higher feeding and activity-dependent energy expenditure (Figure 3G). LeedKO animals also remained relatively glucose tolerant, normo-insulinemic and insulin sensitive (Figure 3H–J). Importantly, this resistance to diet-induced obesity and metabolic syndrome in LEedKO mice was completely abolished in parallel sets of animals that underwent concomitant Ihh immunoneutralization using the 5E1 antibody (Figure 3K–M). Thus, hepatocyte-specific Eed/PRC2 ablation protects animals from obesity and diabetes via Ihh.

### 3.6 Eed/PRC2 controlled Indian hedgehog induces white adipose tissue browning

**Figure 4:**
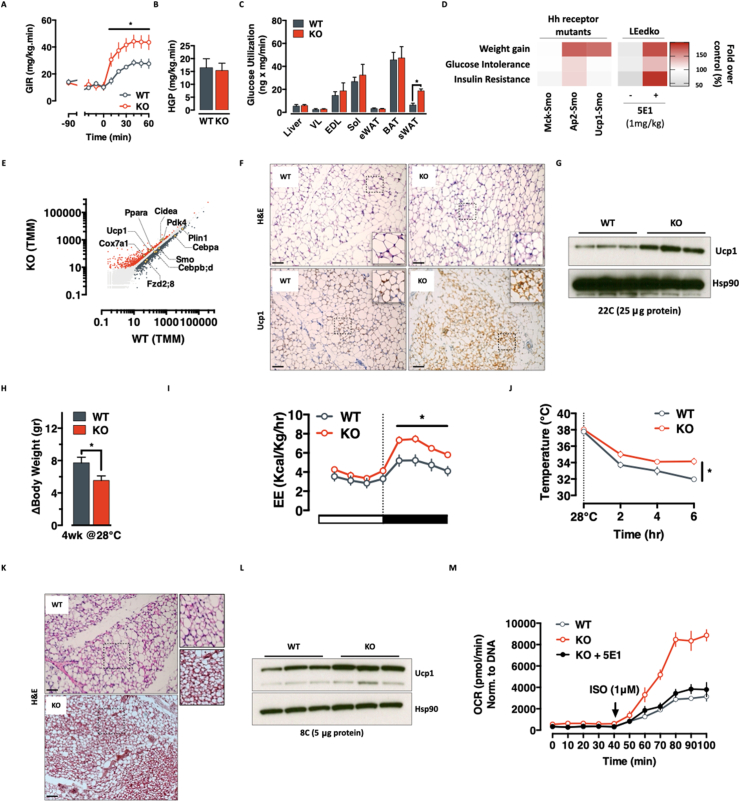
**Eed-controlled Ihh induces white adipose tissue browning**. **A-C.** Glucose Infusion Rate (GIR - **A**), Hepatic Glucose Production (HGP - **B**) and tissue specific glucose uptake (**C**) in WT and LEedKO mice during an euglycemic-hyperinsulinemic clamp (Data expressed as mean ± SEM; n = 6–8 ∗ = *p-value* < 0.05). **D.** Weight gain, glucose intolerance and insulin resistance in tissue-specific (mck = muscle, ucp = brown adipose and ap2 = white adipose) Smo knockout mice. HFD-fed LEedKO mice with and without Ihh immunoneutralization are used as reference. Data is expressed as fold over respective WT control (%). **E.** Scatter plot of TMM-normalized read counts in WT and LEedKO scWAT samples. Up-regulated (red) and down-regulated (gray) genes are highlighted, together with key regulators of browning and hedgehog signalling. **F.** IHC representation of scWAT browning in LEedKO mice. Top = H&E representation of multilobulated adipocytes; bottom = UCP1 staining. **G.** Western Blot representation of UCP1 protein expression in WT and LEedKO scWAT at standard mouse house temperature (22 °C). **H–I.** Body weight gain (**H**) and energy expenditure (**I**) in WT and LEedKO mice exposed to thermoneutrality (28 °C) for four weeks (Data expressed as mean ± SEM; n = 12 ∗ = *p-value* < 0.05). **J-L.** Body temperature (**J**) H&E staining of scWAT (**K**) and Western blot representation of UCP1 protein expression (**L**) in WT and LEedKO mice after 6 h of cold exposure (8 °C) (Data expressed as mean ± SEM; n = 8–12 ∗ = *p-value* < 0.05). **M.***Ex-vivo* analysis of Isoprotherenol-induced fatty acids oxidation in scWAT pieces from WT, LEedKO, and LEedKO mice pre-treated with the soluble α-Hh antibody 5E1 (Data expressed as mean ± SEM; n = 8–12 ∗ = *p-value* < 0.05).

**Supplementary Figure 4:**
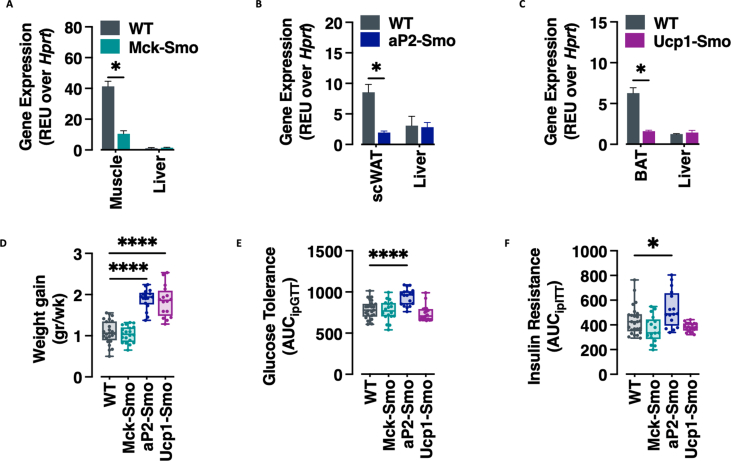
related to figure 4. **Tissue-specific Smo knockout models**. **A-C. qRT-PCR-based Smo gene expression level in muscle (A)**, white (B) and brown (C) adipose-specific knockouts. Liver reported as non-affected control tissue. D-F Body weight gain (D), glucose tolerance (E) and insulin resistance (F) in tissue-specific Smo knockout models. Significance calculated by two-tailed t-test. ∗ = *p*-value <0.05; ∗∗∗∗ = *p*-value <10^−4^.

The data above suggested increased insulin senstivity in LEedKO animals. We validated this notion using gold standard euglycaemic-hyperinsulinemic clamps, finding indeed, that LEedKO mice required higher glucose infusion rates during clamp (Figure 4A). Surprisingly, concomitant glucose tracing measurements revealed no change in liver glucose uptake or production (Figure 4B–C), but rather, a marked increase in subcutaneous white adipose tissue glucose uptake (Figure 4C). To probe the relevance of physiological hedgehog signalling on metabolic control, we knocked out the canonical Hedgehog receptor Smoothened (Smo) in skeletal muscle (Supp. Fig. S4A), white (Supp. Fig. S4B) and brown (Supp. Fig. S4C) adipose tissues. Interestingly, white adipose knockout specifically led to increased adiposity and impaired glucose metabolism (Figure 4D and S4D-F), suggesting scWAT as the major contributor to the observed improvements in LEedKO metabolic homeostasis.

**Supplementary Figure 5:**
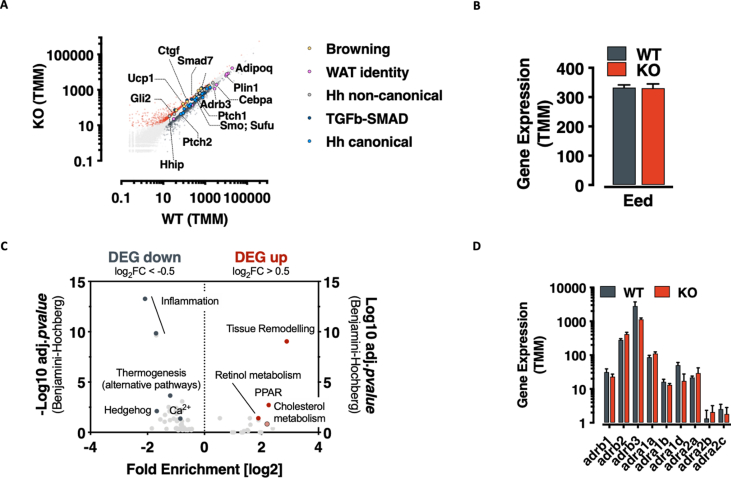
related to figure 4. **scWAT transcriptomic analysis**. **A-B.** RNA-seq based expression levels of Hedgehog signalling pathway components (**A**) and Eed (**B**) in WT and LEedKO scWAT. **C.** KEGG-based pathway analysis of differentially expressed genes between WT and LEedKO scWAT. **D.** RNA-seq based expression levels of adreno-receptor genes in WT and LEedKO scWAT.

Consistent with this idea, RNAseq analysis of LEedKO versus Control scWAT showed increased expression of brown adipocyte markers (Figure 4E) suggesting the metabolic phenotype might be driven by activation of a thermogenic program. No evidence was found of altered expression of either Hedgehog signalling components (Supp. Fig. S5A), or of Eed (Supp. Fig. S5B) arguing against leakiness in the genetic intervention (see also supp. Fig. S2G) or activation of the canonical hedgehog signalling pathway. Pathway analysis revealed enrichment of ontology terms related to tissue development, cellular differentiation and lipid metabolism (Supp. Fig. S5C). By immunohistochemistry and Western blot analyses, we obeserved increased Ucp1 protein expression specifically in multilobulated small adipocytes (Figure 4F–G). Thus, LEedKO mice exhibit what appears to be a pronounced beiging of scWAT under basal conditions. Mechanistically – and based on scWAT RNA-seq data – this beiging is independent from canonical Hh-signalling and β-adrenergic thermogenic programming (Supp. Fig. S5A and S5D). Conversely, the transcriptional activation of the Gli2-Tgfβ-Smad axis (Supp. Fig. S5A) suggests the adipocytes thermogenic reprogramming occurring in a Tgfβ/Hh-responsive stromal environment, rather than a classical beige adipocytes recruitment.

To test thermogenic function directly, we subjected cohorts of LEedKO and Control animals to either thermoneutrality or cold challenge. Upon thermoneutrality, LEedKO mice proved resistant to weight gain (Figure 4H) and remained more metabolically active (Figure 4I). When challenged with acute cold exposure, LEedKO animals showed a more robust ability to defend their body temperatures (Figure 4J). Postmortem analysis revealed exceptional scWAT beiging in LEedKO animals with increased Ucp1 protein expression and nearly 100% of scWAT adipocytes being multilobulated (Figure 4K–**L**). In line with these findings, ex-vivo fatty acid oxidation assays in scWAT tissue pieces showed that LEedKO scWAT responded better to beta-adrenergic stimulation (Figure 4M), without any detectable difference in the expression of the adrenoreceptor genes themselves (Supp. Fig. S5D). Again, and importantly, these phenotypes were Hh-dependent, as evidenced by LEedKO scWAT adipose tissue no longer showing differences when animals were pre-treated with the Hh-immunoneutralizing 5E1 antibody (Figure 4M). These data indicate that LEedKO mice have more thermogenic and functional scWAT depots. Collectively, these findings identify a novel PRC2-buffered, VLDL-Ihh-based endocrine axis important for metabolic health.

## 4 Discussion

Endocrine signaling is the primary basis for coordinated multi-tissue metabolic control. The liver contributes to this process by receiving numerous signals and adjusting its function, as well as contributing its own ‘hepatokine’ signals – liver secreted hormones [48]. Hepatokines actively contribute to adipose tissue metabolic plasticity, including by promoting thermogenesis, regulating proliferation and adipogenesis, attenuating inflammation and fibrosis, suppressing excess free fatty acids (FFA) fluxes, and improving insulin sensitivity [[49], [50], [51]]. Here, we add Indian Hedgehog (Ihh) to this unique set of metabolic regulators. We show that Ihh secretion occurs in response to fast-feeding transitions (Figure 1) and that this secretory dynamic is largely blunted with age and obesity (Figure 1 – more than 3x reduction in refed-induced Ihh secretion in both old and HFD-fed mice**)**. We show that Ihh regulates whole body glucose homeostasis by inducing a thermogenically competent beiging response in subcutaneous white adipose (Figure 3, Figure 4). The exact mechanism by which Ihh induces WAT browning is not known, but our prior work suggests that this likely results via both canonical and non-canonical means [18,19], and independent of excess adrenergic signalling (Supp. Fig. S5) [18].

Our findings in a small yet extensively studied group of male volunteers suggest that this axis is at least partially conserved in humans. They confirm previous findings of Ihh loading onto VLDL [8], and identify clear correlations between VLDL-Ihh levels and metabolic health (Figure 1). Altogether, these findings suggest – with the limitation of a small and male-only cohort – Ihh is a novel effector of whole body metabolic flexibility [52], and suggest even that the metabolic inflexibilities associated with age and elevated BMI depend in part on VLDL-Ihh dynamics [[53], [54], [55], [56]]. Although fast-feeding cycles in mice are not easily extrapolated to human physiology, clinical sampling after overnight fasting in humans represent a snapshot of a individual physiological response to fasting (generally between 10 and 14 h).

Our hepatocyte-specific Eed-KO strategy highlights a potential importance of chromatin dynamics in regulation of this novel axis. We show that the *Ihh* promoter is embedded in bivalent chromatin (Figure 2) and that the locus is highly responsive to Polycomb Repressive Complex 2 (PRC2) dosage (Figure 2). As critical regulator of transcription at bivalent genes, PRC2 guards cell identity [[27], [28], [29],^57^] by preventing re-expression of developmental genes [29,58], and it is therefore not surprising that its dysregulation has been implicated in cancer [59]. Notably – and worth further investigation – Eed knockout hepatocytes showed no evidence in our hands of either cancer, or lost cell identity (Table S2) despite evidenced upregulation of developmental genes (Supp. Fig. S3) (Table S2). Our hepatocyte-specific Eed-KO strategy restricts the effects of PRC2 loss-of-function to hepatocytes. Nevertheless, the upregulation of circulating hedgehog ligands (in particular Ihh) might have systemic effects – worth further and careful investigations, given the oncogenic nature of the Hedgehog pathway and the long-term safety concerns of modulating PRC2 or Hedgehog activity – that we have not detected with our gross pathology and metabolic phenotyping pipeline.

Using data from Halpern et al. [60] as well as our own primary data, we showed that both genomic bivalency as well as *Ihh* transcription are zonated towards the portal node (Supp. Fig. S3). The phenomenon of liver “zonation” [[61], [62], [63]] describes a radial axis that forms lobular functional gradients throughout the liver. Delimited by the portal node (through which oxygen-rich blood enters) and the central vein (through which blood drains out), zonation effectively comprises a functional gradient with energy demanding processes (e.g., gluconeogenesis, beta-oxidation, cholesterol biosynthesis) concentrated at the portal node [60] and less energy demanding processes (glycolysis, bile acids synthesis and xenobiotic metabolism) at the central vein [60]. Bivalent chromatin in adult liver is a feature of pro-regenerative genes, which, while residing in a relatively quiescent genomic environment, have to be rapidly activated upon liver damage [64]. Interestingly, canonical Hedgehog signalling pathway activation is required for liver regeneration, regulating capillarisation, hepatic stellate cell fate, fibrosis and cancer [65].

In summary, the data presented here indicate that PRC2 activity in liver controls glucose homeostasis, via a dynamic repression of the novel, Ihh-centric, endocrine axis. Our findings show that the PRC2-Ihh axis improves metabolic control through adipose tissue browning, is inversely correlated to metabolic health in mice and humans, and therefore might serve as a potential therapeutic target for type 2 diabetes and obesity.

## CRediT authorship contribution statement

R.Teperino., M.Adamovà., S.Aljabali., S.Pai., R.Gerlini., I.Paez-Perez., M.Matz-Soja., S.Heyne., E.Schleicher and M.Basilicata: Methodology, Investigation, Formal analysis, Data curation. R.Teperino., JA. Pospisilik: Conceptualization, Funding acquisition, Project administration, Writing - original draft, Writing - review & editing. M.Hrabe de Angelis: Writing - review & editing, Funding acquisition. A.Lempradl, E.Schleicher, R.Gebhardt, HU.Häring: Writing - review & editing.

## Declaration of competing interest

The authors declare no conflict of interest.

## Data Availability

Data will be made available on request.
